# The Structure and Optical Properties of Luminescent Europium Terephthalate Antenna Metal–Organic Frameworks Doped by Yttrium, Gadolinium, and Lanthanum Ions

**DOI:** 10.3390/molecules29153558

**Published:** 2024-07-28

**Authors:** Oleg S. Butorlin, Anna S. Petrova, Yulia N. Toikka, Ilya E. Kolesnikov, Sergey N. Orlov, Mikhail N. Ryazantsev, Nikita A. Bogachev, Mikhail Yu. Skripkin, Andrey S. Mereshchenko

**Affiliations:** 1 Saint Petersburg State University, 7/9 Universitetskaya emb., Saint Petersburg 199034, Russia; olbuse@mail.ru (O.S.B.); an.petra04.floreo@gmail.com (A.S.P.); helmi24@mail.ru (Y.N.T.); ilya.kolesnikov@spbu.ru (I.E.K.); orlov.s.n.1989@yandex.ru (S.N.O.); mikhail.n.ryazantsev@gmail.com (M.N.R.); n.bogachev@spbu.ru (N.A.B.); skripkin1965@yandex.ru (M.Y.S.); 2 Institute of Nuclear Industry, Peter the Great St. Petersburg Polytechnic University (SPbSU), 29, Polytechnicheskaya Street, Saint Petersburg 195251, Russia; 3 Nanotechnology Research and Education Centre RAS, Saint Petersburg Academic University, ul. Khlopina 8/3, Saint Petersburg 194021, Russia

**Keywords:** metal–organic framework, luminescence, rare earth, europium, yttrium, gadolinium, lanthanum, antenna effect

## Abstract

New heterometallic antenna terephthalate MOFs, namely, (Eu_x_M_1−x_)_2_bdc_3_·4H_2_O (M = Y, La, Gd) (x = 0.001–1), were synthesized by a one-step method from aqueous solutions. The resulting compounds are isomorphic to each other; the crystalline phase corresponds to Ln_2_bdc_3_∙4H_2_O. Upon 300 nm excitation to the singlet excited state of terephthalate ions, all compounds exhibit a bright red emission corresponding to the of ^5^D_0_–^7^F_J_ (J = 0–4) f-f transitions of Eu^3+^ ions. The Eu(III) concentration dependence of the photophysical properties was carefully studied. We revealed that Gd-doping results in photoluminescence enhancement due to the heavy atom effect. To quantitatively compare the antenna effect among different compounds, we proposed the new approach, where the quantum yield of the ^5^D_0_ formation is used to characterize the efficiency of energy transfer from the ligand antenna to the Eu^3+^ emitter.

## 1. Introduction

Metal–organic frameworks (MOFs) represent a large class of crystalline materials defined as porous networks, consisting of metallic ions or clusters linked together by organic multidentate ligands. Due to their well-defined crystallinity, porosity, high stability, and wide diversity of structures and topologies, these materials have attracted considerable attention in the past two decades. Rare-earth element (REE) metal–organic frameworks (MOFs) are of particular interest due to their unique luminescence properties significantly determined by the type of lanthanide ion. Thus, REE-MOFs have been revealed as promising candidates for light-emitting materials, sensors, multimodal image contrast agents, catalysts, and analytes to reveal the hazardous substances in food and enviroment [1,2,3,4,5,6,7]. The f-f transitions are forbidden by selection rules, which results in inefficiency of direct excitation of lanthanide ions. This problem can be overcome by using the energy transfer from the excited ligand to the lanthanide ion, which is called the “antenna effect” [8,9]. The organic ligands, which are used as “antenna” compounds, have high UV absorption coefficients, easily coordinate with REE ions, and efficiently transfer energy to REE ions. The mechanism of the antenna effect can be explained as follows. Upon photon absorption, the ligand in the ground singlet state (S_0_) is promoted to the singlet excited state (S_1_, S_2_, etc.), followed by fast internal conversion to a lower excited energy level (S_1_). The excited singlet state can then either (i) return to the ground state (S_1_→S_0_) by internal conversion and fluorescence or (ii) undergo an intersystem crossing to the triplet state. Through internal conversion, the ligand reaches the lowest triplet level electronic state, T_1_, followed by the energy transfer to the REE ion [10]. In Eu-based antenna complexes, the quantum yield of luminescence from the europium ion depends on the relative energy level of the ligand triplet state and the atomic level of the Eu^3+^ ion. Energy transfer has been found to occur from the lowest energy triplet state of the ligand T_1_ to the ^5^D_J_ (J = 0–3) level of the Eu^3+^ ion followed by internal conversion to ^5^D_0_. The radiative transitions ^5^D_0_–^7^F_J_ (J = 0–4) to the term of ground state ^7^F of the Eu^3+^ ion correspond to the resulting photoluminescence of such antenna complexes. For the most efficient energy transfer, the difference in energy between the ligand triplet state and the ^5^D_J_ level of Eu^3+^ should be approximately 2500–4000 cm^−1^ [11]. However, it has also been proposed that energy transfer can occur from a singlet excited state as well [12,13]. For example, in a study by Shinji Miyazaki et al., the possibility of two energy transfer pathways was revealed from both the triplet and singlet levels for the Eu(hfa)_3_(DPPTO)_2_ complex (hfa—hexafluoroacetylacetonate, DPPTO—2-diphenylphosphoryltriphenylene) [14].

The simultaneous presence of both luminescent and nonluminescent REE ions, such as Sc^3+^, Y^3+^, La^3+^, Gd^3+^, Lu^3+^, can significantly affect the structural and photophysical properties of these compounds. The structural properties of heterometallic REE-MOFs have been investigated in several studies. It has been found that at low concentrations of the luminescent lanthanide ions, substitution occurs isomorphically, without changing the crystalline structure. However, it has been observed that in some compounds, the structure changes as concentration increases. For example, Jarley Nascimento et al. showed that compounds Gd_1−x_Eu_x_(1,4-bdc)_3_(dmf)_2_(H_2_O)_n_ (bdc—benzenedecarboxylate, dmf—dimethylformamide; x = 0.01, 0.03, 0.05, 0.07, 0.09) are isostructural with [Eu_2_(1,4-bdc)_3_(dmf)_2_(H_2_O)] at the Eu^3+^ content between 1 and 7 at. % (at. %—the relative atom content of a certain lanthanide to all lanthanide atoms). However, at the Eu^3+^ concentration of 9 at. %, the compound was isostructural with [Eu_2_(1,4-bdc)_3_(dmf)_2_(H_2_O)_2_] [15]. In our previous research, we found that the compound (Eu_x_Lu_1−x_)_2_(1,4-bdc)_3_∙nH_2_O forms different crystal structures depending on the concentration of the Eu^3+^ ions. At the Eu^3+^ concentration range of 6–100 at. %, the samples were found to be isostructural with Ln_2_(1,4-bdc)_3_∙4H_2_O. However at the Eu^3+^ concentration of 0–2 at. %, the samples were isostructural with the Ln_2_bdc_3_. At the Eu^3+^ concentration between 3 and 5 at. %, both the Ln_2_bdc_3_∙4H_2_O and the Ln_2_(1,4-bdc)_3_ phases were observed in the samples [16]. The changes in the crystal structure can significantly affect the optical properties of compounds, such as the fine structure of emission spectra, quantum yields, and lifetime values.

Meanwhile, the optical properties of the heterometallic MOFs also depend on the concentration of the luminescent ion in the case of isomorphic substitution of the REE ion by the luminescent ion in the whole concertation range where the single crystalline phase is formed. However, very few works have studied such a concentration dependence. Thus, Utochnikova et al. studied the optical properties of heterometallic solid solutions of (Tb_x_Y_1−x_)_2_(1,4-bdc)_3_(H_2_O)_4_ and Eu_x_Gd_1−x_(dbm)_3_(phen) (dbm—dibenzoylmethanate, phen—o-phenantroline) MOFs. The steep quantum yield rise was observed at the low concentrations of Eu^3+^ or Tb^3+^ ions. Then, in the range of 20–100 at. % of Tb^3+^ for (Tb_x_Y_1−x_)_2_(1,4-bdc)_3_(H_2_O)_4_ and 10–100 at. % of Eu^3+^ for Eu_x_Gd_1−x_(dbm)_3_(phen), the quantum yield does not depend on the concentration of the luminescent ions. It has also been observed that lifetimes decrease with increasing concentration of the luminescent ion [17].

We note that the majority of studies focus on compounds with a single or a few concentrations of the luminescent lanthanide ion [17,18,19] or a narrow range of concentrations [15]. The properties of the synthesized compounds have been studied incompletely, and, therefore, at the moment, we have limited information about the mechanism of the dopant concentration effect on photophysical properties of heterometallic REE-MOFs.

In this article, the photophysical properties (photoluminescence decay time constants, radiative, nonradiative, and total decay rates, quantum efficiencies, and formation quantum yields of the ^5^D_0_ level; photoluminescence quantum yields, asymmetric ratios) of REE-MOFs of solid solutions of heterometallic (Eu_x_M_1−x_)_2_(1,4-bdc)_3_∙4H_2_O MOFs (M = Y, La, Gd) were studied in detail in a wide concentration range of the Eu^3+^ ion (0.1–100 at. %).

## 2. Results

### 2.1. Structure and Morphology

**Figure 1:**
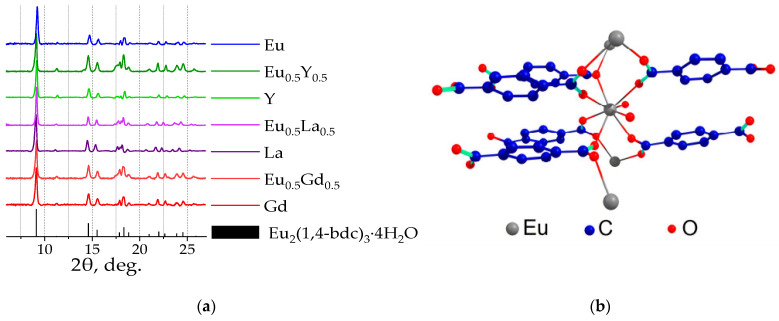
The XRD patterns of selected (Eu_x_M_1−x_)_2_bdc_3_·4H_2_O (M = Gd, La, Y; x = 0, 0.5, 1) and the simulated XRD pattern of Eu_2_(1,4-bdc)_3_·4H_2_O single-crystal structure were taken from ref. [20] (**a**). The crystal structure of Eu_2_(1,4-bdc)_3_·4H_2_O (**b**).

The phase composition of (Eu_x_M_1−x_)_2_(1,4-bdc)_3_·nH_2_O (M = Y, Gd, La) with a Eu^3+^ concentration from 0 to 100 at. % was studied using powder X-ray diffraction (PXRD). The experimental PXRD patterns at. %of the synthesized materials are presented in Figure 1a and Figure S1 (Supplementary Materials). The positions of the diffraction maxima in the PXRD patterns indicate that all the synthesized compounds correspond to the Ln_2_(1,4-bdc)_3_·4H_2_O crystalline phase (Ln = Ce − Yb) [20], and no additional peaks were observed. In the Ln_2_(1,4-bdc)_3_·4H_2_O structure (Figure 1b), the octacoordinated lanthanide ions are bound to the two water molecules and six different terephthalate ions through the oxygen atoms.

**Figure 2:**
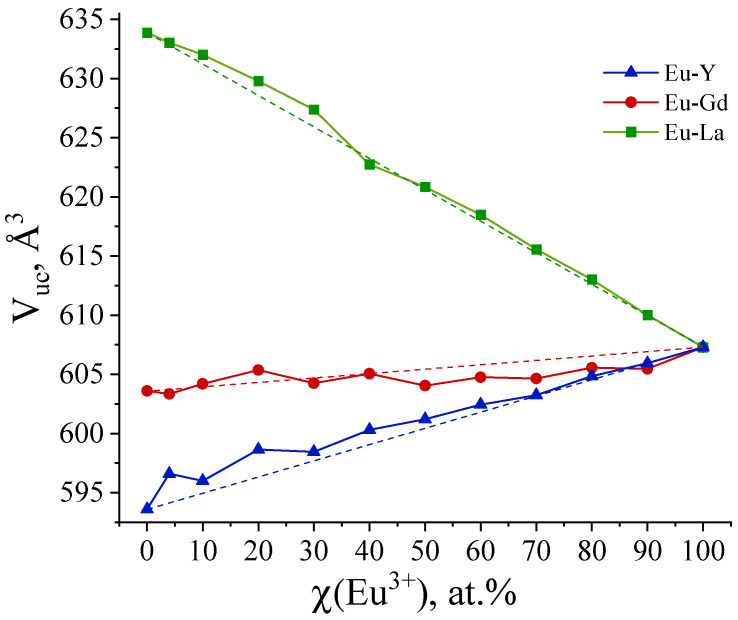
Eu^3+^ concentration dependence of unit cell volume (V_uc_) refined for (Eu_x_M_1−x_)_2_(1,4-bdc)_3_·4H_2_O (M = Gd, La, Y).

The refinement of the unit cell parameters and the calculation of the unit cell volumes were performed for the selected samples over the Eu^3+^ concentration range between 0 and 100 at. %. Unit cell parameters (Table S1, Supplementary Materials) were refined using UnitCell software [21]. This program can retrieve unit cell parameters from diffraction data using a least-squares method from the positions of the indexed diffraction maxima of the PXRD patterns (Pawley method [22]). The Eu^3+^ concentration effect on the unit cell volumes is shown in Figure 2. For the (Eu_x_La_1−x_)_2_(1,4-bdc)_3_·4H_2_O compounds, the increase in La^3+^ content leads to increased unit cell volumes due to a higher ionic radius of La^3+^ ions (1.160 Å, the coordination number is eight) than the ionic radius of Eu^3+^ ions (1.066 Å) [23].

The ionic radius of the Gd^3+^ ion (1.053 Å) is close to that of Eu^3+^. Therefore, the unit cell parameters do not change significantly in the (Eu_x_Gd_1−x_)_2_(1,4-bdc)_3_·4H_2_O series. The Y^3+^ ion (0.977 Å) is less than Eu^3+^; therefore, substitution of Eu^3+^ by the Y^3+^ ion results in a decrease in the unit cell volumes in the (Eu_x_M_1−x_)_2_(1,4-bdc)_3_·4H_2_O series. In general, the dependence of unit cell volume obeys Vegard’s law [24], with a slight deviation from linearity that falls within the error limits.

**Figure 3:**
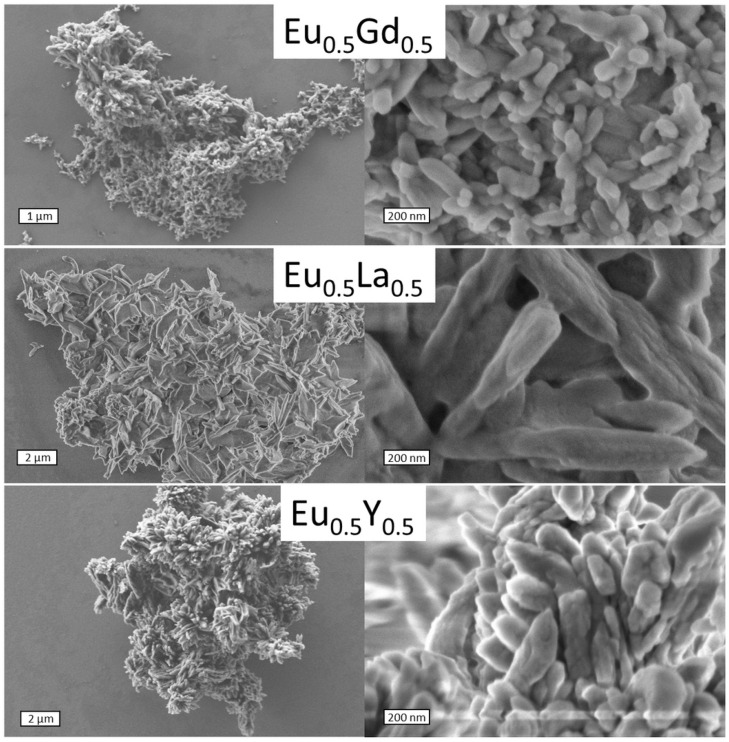
SEM images of (Eu_0.5_M_0.5_)_2_(1,4-bdc)_3_∙4H_2_O (M = Gd, La, Y).

Scanning electron microscopy (SEM) was used to reveal the particle morphology and the porosity of the selected synthesized materials, namely, (Eu_0.5_M_0.5_)_2_(1,4-bdc)_3_·4H_2_O (M = Y, La, Gd). The resulting compounds had a distinct porous structure, as can be seen from the SEM images in Figure 3. On average, the particles are between 5 and 20 µm in size, and the pore diameter ranges from 20 to 150 nanometers. This observation confirms that the synthesized compounds are MOFs according to the definition of IUPAC [25]. Heterometallic europium terephthalates doped with yttrium and gadolinium formed spindle-shaped particles, while those with lanthanum formed flake-like particles.

### 2.2. IR Spectroscopy

**Figure 4:**
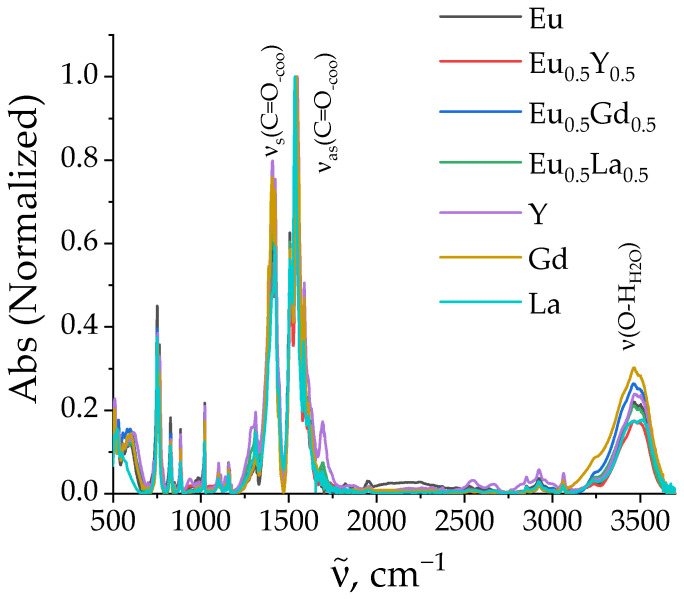
FTIR spectra of (Eu_0.5_M_0.5_)_2_(1,4-bdc)_3_·4H_2_O (M = Y, Gd, La) and M_2_(1,4-bdc)_3_·4H_2_O (M = Y, Gd, La, Eu).

To reveal the doping effect on the vibrational structure of the ligands, we measured the IR spectra of the selected samples of heterometallic (Eu_0.5_M_0.5_)_2_(1,4-bdc)_3_·4H_2_O (M = Y, Gd, La) and homometallic M_2_(1,4-bdc)_3_·4H_2_O (M = Y, Gd, La, Eu) terephthalates (Figure 4). The broad band with the maximum at about the 3500 cm^−1^ region corresponds to the O-H stretching vibrations of the water molecules coordinated to the metal. The multiple narrow bands in the 1270–1470 and 1470–1800 cm^−1^ regions correspond to the symmetric and asymmetric stretching vibrations of the carboxylic -COO group of the terephthalate ion, respectively. The data obtained are consistent with the data in the literature obtained for REE terephthalates [26,27]. The spectral shape, including the fine structure of the absorption bands and the position of the absorption maxima, is almost identical for the studied compounds, which indicates the similar structure of these terephthalates. This conclusion is in agreement with PXRD data, demonstrating that all the synthesized compounds have the same crystalline phase, namely, Ln_2_(1,4-bdc)_3_·4H_2_O.

### 2.3. Thermogravimetric Analysis

**Figure 5:**
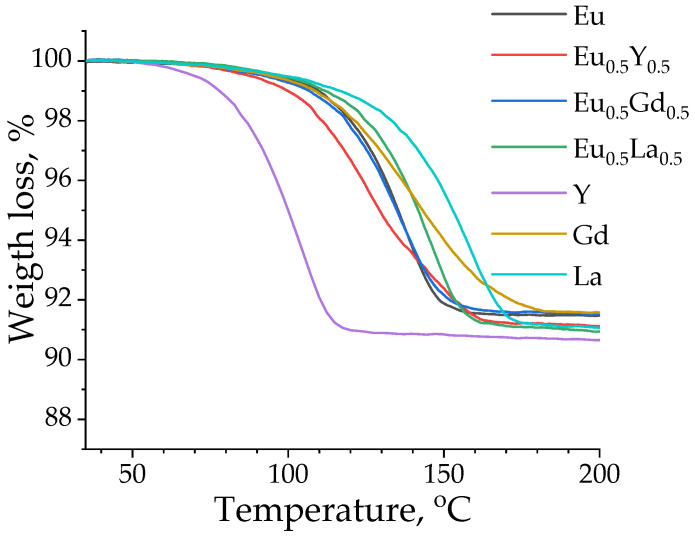
TGA curves of selected heterometallic (Eu_0.5_M_0.5_)_2_(1,4-bdc)_3_·4H_2_O (M = Y, Gd, La) and homometallic M_2_(1,4-bdc)_3_·4H_2_O (M = Y, Gd, La, Eu) terephthalates measured in the temperature range of 35–200 °C.

Thermogravimetric analysis (TGA) was performed for selected heterometallic (Eu_0.5_M_0.5_)_2_(1,4-bdc)_3_·4H_2_O (M = Y, Gd, La) and homometallic M_2_(1,4-bdc)_3_·4H_2_O (M = Y, Gd, La, Eu) terephthalates in the temperature range of 35–200 °C (Figure 5). Weight loss between 8.5 and 9.3% was observed at a temperature of 120–180 °C for all measured samples. As previously reported [28], weight loss in this temperature range may be associated with the dehydration of compounds, leading to the formation of anhydrous terephthalates with a general formula of M_2_(1,4-bdc)_3_. The weight loss of 8.5–9.3% corresponds to the elimination of 3.8–4.1 water molecules from the initial terephthalates, which is in agreement with the PXRD data, showing that all studied materials are formed in the Ln_2_(1,4-bdc)_3_∙4H_2_O crystalline phase.

### 2.4. Luminescent Properties

**Figure 6:**
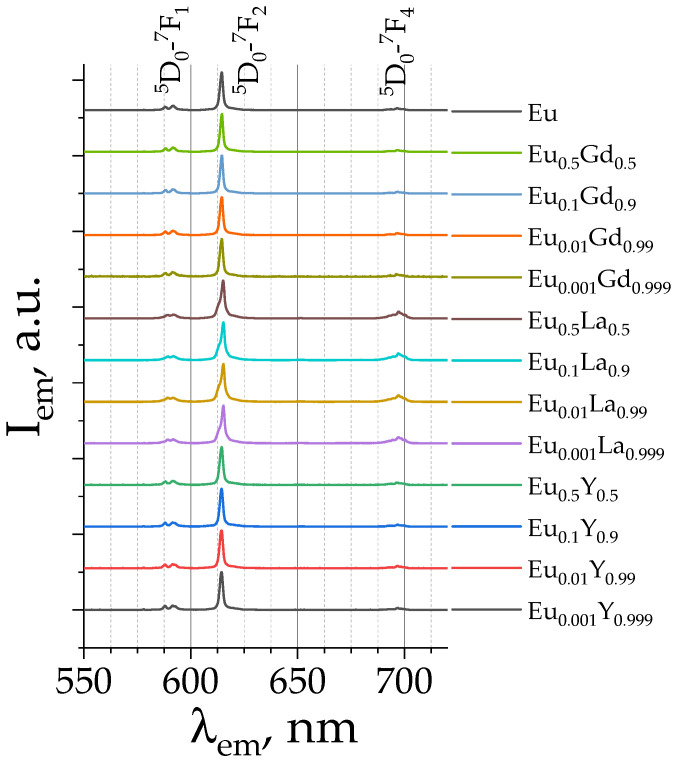
The normalized emission spectra of (Eu_x_M_1−x_)_2_(1,4-bdc)_3_∙4H_2_O (M = Gd, La, Y) at selected Eu^3+^ concentrations (given in legend) upon 300 nm excitation.

For all synthesized compounds, emission spectra were measured upon 300 nm excitation into the S_n_ singlet state of the terephthalate ion. Figure 6 shows the normalized emission spectra of selected samples with different Eu^3+^ content ((Eu_x_M_1−x_)_2_(1,4-bdc)_3_·4H_2_O (M = Y, La, Gd) (x = 0.001, 0.01, 0.1, 0.5, 1)). The emission spectra of all studied materials are given in Figure S2 (Supplementary Materials). All emission spectra contain the same narrow bands, which correspond to the ^5^D_0_–^7^F_J_ (J = 1, 2, 4) transitions of Eu^3+^: ^5^D_0_–^7^F_1_ (587.9 and 591.6 nm), ^5^D_0_–^7^F_2_ (614 nm), and ^5^D_0_–^7^F_4_ (696 nm).

^5^D_0_–^7^F_3_ transitions are also present in emission spectra, but they were not observed due to their weak intensity. The presence of Eu^3+^ f-f bands in the emission spectra upon excitation to the S_n_ singlet state of the terephthalate ion clearly reveals an antenna effect. Thus, the terephthalate ion absorbs UV radiation followed by efficient energy transfer to the luminescent lanthanide ion. Upon the excitation, the terephthalate ion is promoted into the S_n_ state, followed by the fast internal conversion to the S_1_ state. Due to the heavy atom effect caused by the lanthanide atom, the S_1_ state efficiently undergoes intersystem crossing to the T_1_ triplet electronic excited state. The T_1_ state of the terephthalate ion is close in energy to the ^5^D_1_ energy level of the Eu^3+^ ion [11,17]. Therefore, an efficient energy transfer occurs from the sensitizer to the luminescent lanthanide ion. The ^5^D_1_ level of Eu^3+^ then undergoes an internal conversion to the ^5^D_0_ state, followed by the emission to the ^7^F_J_ (J = 1, 2, 4) lower-lying energy levels.

The shape of the emission spectra for the Y-, Gd-, and La-doped compounds is identical to that of the pure europium terephthalate emission spectrum (Figure S3, Supplementary Materials), implying the same coordination environment of Eu^3+^ in the solid solutions studied. This observation agrees with the PXRD data, which show the presence of the same crystalline structure, (Eu_x_M_1−x_)_2_(1,4-bdc)_3_·4H_2_O (M = Y, Gd, La), among the studied series. However, the peak intensity of emission spectra depends on the concentrations of europium ions in the studied solid solutions due to different photoluminescence quantum yields, which will be discussed further.

**Figure 7:**
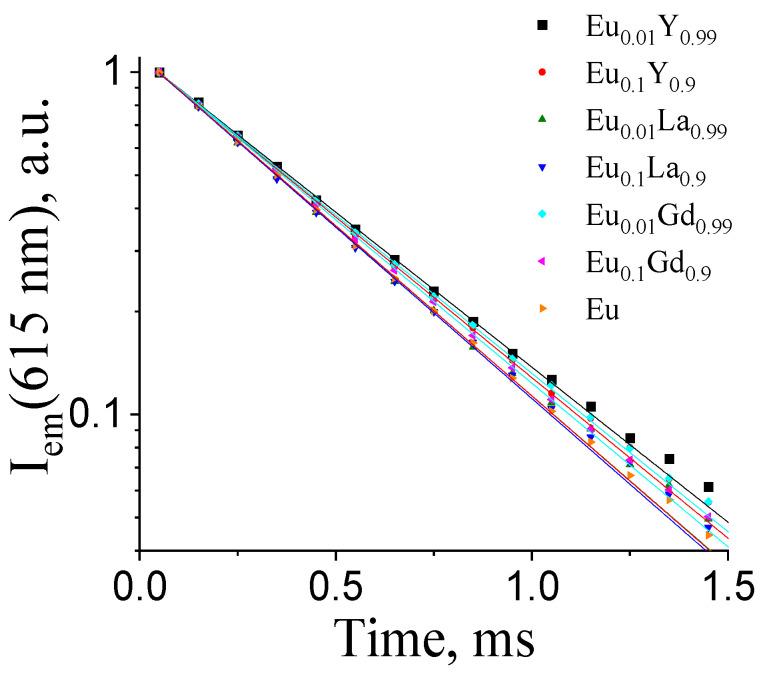
The 615 nm photoluminescence decay curves of (Eu_x_M_1−x_)_2_(1,4-bdc)_3_·4H_2_O (M = Y, La, Gd; x = 0.01, 0.1).

The photoluminescence decay curves of the (Eu_x_M_1−x_)_2_(1,4-bdc)_3_·4H_2_O phosphors monitored at 615 nm (^5^D_0_–^7^F_2_ transition) are presented in Figure 7 (λ_ex._ = 300 nm). The decay curves were fitted by a single exponential function:

(1) $$I=I_{0}\cdot e^{\frac{-t}{\tau }}$$

where *τ* is the observed ^5^D_0_ lifetime (Table 1).

Figure 7 and Table 1 present the photoluminescence decay curves and lifetime data for the selected concentrations of Eu^3+^ ions in (Eu_x_M_1−x_)_2_(1,4-bdc)_3_·4H_2_O (M = Gd, La, Y) (x = 0.01, 0.1, and 1) among the whole concentration range. As can be seen from Table 1, all the samples have similar lifetimes. This allows us to assume that the ^5^D_0_ lifetime of the Eu^3+^ ion almost does not depend on the Eu^3+^ content in the 1–100 at. % concentration range of Eu^3+^.

**Figure 8:**
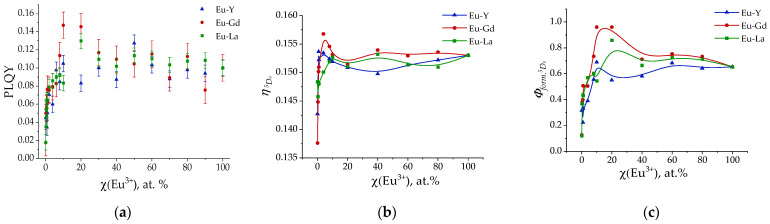
Photoluminescence quantum yield (PLQY) (**a**), quantum efficiency (η) ^5^D_0_ of Eu^3+^ (**b**), and quantum yield of ^5^D_0_ formation (**c**) of (Eu_x_M_1−x_)_2_(1,4-bdc)_3_·4H_2_O (M = Gd, La, Y).

The measured photoluminescence quantum yields (PLQYs) of the (Eu_x_M_1−x_)_2_(1,4-bdc)_3_·4H_2_O are shown in Figure 8a. It should be noted that the ^5^D_0_ lifetimes do not depend significantly on the sample composition and are equal to about 0.45 ms. Meanwhile, the photoluminescence luminescence quantum yields increase with an increase in the Eu^3+^ concentration from 1 to 10 at. % due to the number of luminescence sites increasing. In particular, for the Eu–Y series, there is a rapid increase in the quantum yield up to 10%, after which the PLQY remains within small deviations of values from 10 at. % and reaches a plateau, up to Eu 100%. For the Eu–La series, there is also a rapid increase in the PLQY at low concentrations of Eu up to ~12% and a slight decrease in quantum yield to 10% in the range of Eu concentrations from 20 to 100 at. %. The Eu–Gd series is different from the Eu–Y and Eu–La ones: the PLQY has a maximum at the Eu^3+^ content of 10 at. %. The PLQY of (Eu_0.1_Gd_0.9_)_2_(1,4-bdc)_3_·4H_2_O is equal to 15%, which is 1.5 times greater than that of (Eu_0.1_Y_0.9_)_2_(1,4-bdc)_3_·4H_2_O and (Eu_0.1_La_0.9_)_2_(1,4-bdc)_3_·4H_2_O. Upon further Eu^3+^ content increase, the PLQY of (Eu_x_Gd_1−x_)_2_(1,4-bdc)_3_·4H_2_O decreases, whereas the PLQY of (Eu_x_Y_1−x_)_2_(1,4-bdc)_3_·4H_2_O and (Eu_x_La_1−x_)_2_(1,4-bdc)_3_·4H_2_O stays about the same (10%).

Luminescence decay is determined by both radiative and nonradiative processes. Radiative decay rate is determined by dipole transition strength and local-field correction. Nonradiative processes include multiphonon relaxation, quenching on impurities, and cooperative processes such as cross-relaxation and energy migration, the influence of which increases with increasing concentration of europium ions in the solid solution. Radiative and nonradiative decay rates of Eu^3+^-doped phosphors can be calculated from the emission spectrum using the 4f–4f intensity theory [29]. The magnetic dipole ^5^D_0_–^7^F_1_ transition probability $A_{0–1}=A_{MD_{0}}\cdot n_{0}^{3}=\;49\;s^{-1}$. $A_{MD_{0}}$ is the spontaneous emission probability of the magnetic dipole ^5^D_0_–^7^F_1_, 14.65 s^−1^, and $n_{0}$ is the refractive index, 1.5 [30]. Radiative decay rates $A_{0-J}$ (J = 2, 4) of the ^5^D_0_–^7^F_J_ emission transition can be obtained as follows:

(2) $$A_{0–J}=A_{0–1}\cdot \frac{\nu_{0–1}\cdot I_{0–J}}{\nu_{0–J}\cdot I_{0–1}}$$

where *ν*_0–*J*_ and *I*_0–*J*_ are, respectively, the frequency and intensity of the corresponding transition ^5^D_0_–^7^F_J_ in the emission spectrum. The radiative decay rate is the sum of all the $A_{0-J}$ values $A_{\mathrm{Rad}}=A_{0–1}+A_{0–2}+A_{0–4}$. The nonradiative decay rate can be determined using the observed lifetime and the obtained radiative decay rate: $A_{\mathrm{total}}=A_{\mathrm{Rad}}+A_{\mathrm{Nonrad}}=\frac{1}{\tau_{f}}$; the quantum efficiency of the ^5^D_0_ is

(3) ηD0,5=ARadAtotal=n(5D0–7FJ)em.n(5D0)

Knowledge of both the PLQY (Figure 8a) and the quantum efficiency of ^5^D_0_ (Figure 8b) allows us to calculate the quantum yield of the ^5^D_0_ formation. The studied terephthalate solid solutions are antenna complexes, where ^5^D_0_ is populated as a result of the energy transfer from the initial excited terephthalate ion (S_n_(bdc^2−^)→S_1_(bdc^2−^)→T_1_(bdc^2−^)→^5^D_0_(Eu^3+^)). Therefore, the PLQY can be calculated as the direct product of the quantum efficiency of the ^5^D_0_ (ηD0,5) and quantum yield of the ^5^D_0_ formation ($\Phi_{\mathrm{form}.^{5}D_{0}}$):

(4) $$\mathrm{PLQY}=\eta_{{{,}^{5}D}_{0}}\cdot \Phi_{\mathrm{form}.^{5}D_{0}}$$

Therefore, the quantum yield of the ^5^D_0_ formation (Figure 8c) was calculated as follows:

(5) $$\Phi_{\mathrm{form}.^{5}D_{0}}=\frac{\mathrm{PLQY}}{\eta_{{{,}^{5}D}_{0}}}$$

At Eu^3+^ concentrations up to 10 at. %, the quantum yield of the ^5^D_0_ formation increases as a result of the number of luminescence sites increasing. For the Eu–Y series, it remains unchanged up to 100 at. % of Eu^3+^, whereas for the Eu–La and Eu–Gd series, it reaches a maximum at 10 at. % of Eu^3+^ and then decays. The maximum values of quantum yield of the ^5^D_0_ formation decrease in a dopant row of Gd–La–Y. The QY of ^5^D_0_ formation is close to 100%, which indicates a very-high-efficiency energy transfer from the terephthalate antenna to the light-emitting Eu^3+^ ion. We propose that this observation can be explained by the heavy atom effect, which is more pronounced for the Gd^3+^ ion and less pronounced for the Y^3+^ ion.

**Figure 9:**
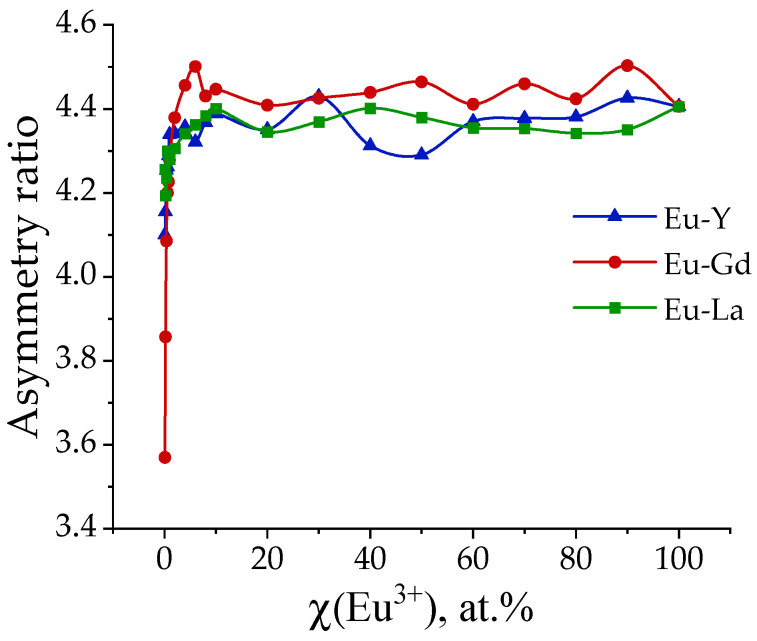
Asymmetric ratios of (Eu_x_M_1−x_)_2_(1,4-bdc)_3_·4H_2_O (M = Gd, La, Y).

The emission spectrum of the Eu^3+^ ion includes a very sensitive forced electric dipole transition ^5^D_0_–^7^F_2_. At the same time, the ^5^D_0_–^7^F_1_ magnetic dipole transition is not sensitive to environmental changes. Changes in the environment can be judged by the asymmetry ratio, which is determined by the ratio between the ^5^D_0_–^7^F_2_ and ^5^D_0_–^7^F_1_ transition intensities. The higher the asymmetry coefficient, the further away the luminescence center is located from the centrosymmetric geometry [31]. The effect of Eu^3+^ ion concentration on local symmetry in samples (Eu_x_M_1−x_)_2_(1,4-bdc)_3_∙4H_2_O MOFs (M = Y, La, Gd) is shown in Figure 9. It is seen that an increase in the concentration of the Eu^3+^ ion leads to the growth of the asymmetry ratio due to distortion of the crystal structure near the luminescent atom. Starting from 10 at. % of Eu^3+^, the asymmetry ratio reaches a plateau and does not change within the margin of error. Thus, the data once again confirm the fact that three-charged REE ions replace each other isomorphically in crystalline substances.

**Figure 10:**
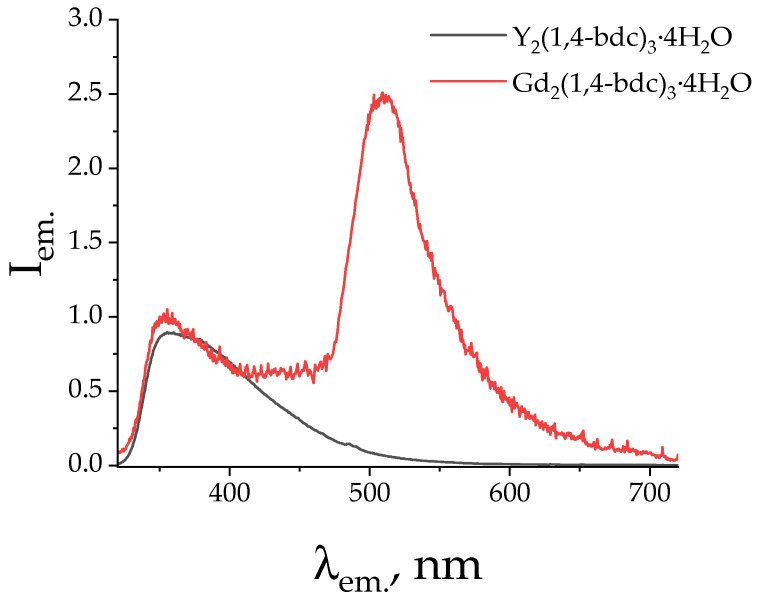
The emission spectra of Gd_2_(1,4-bdc)_3_∙4H_2_O and Y_2_(1,4-bdc)_3_∙4H_2_O upon 300 nm excitation.

The abovementioned results clearly show that the presence of a gadolinium ion leads to the luminescence enhancement in Eu–Gd terephthalates, whereas this effect is almost not pronounced in the Eu–La and Eu–Y series. Thus, (Eu_0.1_Gd_0.9_)_2_(1,4-bdc)_3_∙4H_2_O demonstrates the maximum PLQY among the Eu–Gd series, 15%. The PLQY value is 4–5% higher than that in the Eu–La and Eu–Y series at the same Eu^3+^ ion content (10 at. %). At the higher Eu^3+^ concentrations, the values of PLQY decrease smoothly for the Eu–Gd series, reaching 10% for Eu_2_(1,4-bdc)_3_∙4H_2_O. For the Eu–La and Eu–Y series, the PLQY stays about the same (9–11%) up to Eu^3+^ concentration of 100 at. %. Therefore, we do not observe Eu^3+^ concentration quenching. The Gd-doping effect is also observed for the calculated values of ^5^D_0_ formation quantum yield: for (Eu_0.1_Gd_0.9_)_2_(1,4-bdc)_3_∙4H_2_O, the QY of ^5^D_0_ formation values become close to 100%, which indicates a very-high-efficiency energy transfer from the terephthalate antenna to the light-emitting Eu^3+^ ion. The Gd^3+^ ion has an f^7^ configuration and, therefore, increases the probability (rate) of S_1_-T_1_ intersystem crossing in the terephthalate ion, which is associated with the heavy atom effect manifested by paramagnetic Gd^3+^ ions. To confirm the proposed mechanism, we measured the emission spectra of gadolinium and yttrium terephthalates upon 300 nm excitation into the ^1^ππ band of the terephthalate ion. The prominent increase in the phosphorescence band (510 nm) was observed for Gd_2_(1,4-bdc)_3_∙4H_2_O compared with Y_2_(1,4-bdc)_3_∙4H_2_O, which confirms the increase in the intersystem crossing quantum yield resulting from the increase in the S_1_-T_1_ nonradiative transition rate (Figure 10) as a result of the presence of the Gd^3+^ ion.

## 3. Materials and Methods

Europium (III) chloride hexahydrate, yttrium (III) chloride hexahydrate, gadolinium (III) chloride hexahydrate, and lanthanum (III) chloride hexahydrate were purchased from Chemcraft (Kaliningrad, Russia). Benzene-1,4-dicarboxylic (terephtalic, H_2_(1,4-bdc)) acid (>98%), sodium hydroxide (>99%), nickel(II) chloride hexahydrate (>99%), EDTA disodium salt (0.1 M aqueous solution), and murexide were purchased from Sigma-Aldrich Chemie GmbH (Taufkirchen, Germany) and used without additional purification.

The 0.2 M solutions of EuCl_3_, YCl_3_, GdCl_3_, and LaCl_3_ were prepared and standardized using back complexometric titration with EDTA and nickel chloride solution in the presence of an ammonia buffer (pH ≈ 9). A total of 0.6 mole of sodium hydroxide and 0.3 mole of terephthalic acid were dissolved in distilled water to obtain a 1 L solution of a 0.3 M solution of the disodium terephthalate (Na_2_(1,4-bdc)).

Heterometallic terephthalates with a general formula (Eu_x_M_1−x_)_2_(1,4-bdc)_3_∙4H_2_O (M = Y, Gd, La) were obtained by mixing 0.2 M EuCl_3_, 0.2 M MCl_3_ (M = Y, La, Gd) with 2 mL of 0.3 M Na_2_bdc water solution. EuCl_3_ and MCl_3_ were taken in stoichiometric ratios. The total volume of 0.2 M EuCl_3_ and 0.2 M MCl_3_ solutions was equal to 1 mL. White precipitates of the resulting terephthalates were separated from the reaction mixture using centrifugation (4000× *g*) and washed using deionized water 3 times. The samples of compounds were then dried at 60 °C.

The Eu^3+^/M^3+^ (M = Y, Gd, La) ratios in the heterometallic terephthalates were confirmed using energy-dispersive X-ray spectroscopy (EDX) (EDX spectrometer EDX-800P, Shimadzu, Kyoto, Japan). The Eu/M (M = Y, Gd, La) ratios obtained from EDX were consistent with the expected ratios of Eu^3+^/M^3+^ (M = Y, Gd, La) taken for the synthesis for Eu^3+^ content within 1 at. % accuracy for the most of the samples. The EDX data are provided in the Supplementary Materials in Table S2. X-ray powder diffraction (PXRD) measurements were performed with a D2 Phaser (Bruker, Billerica, MA, USA) X-ray diffractometer using Cu K_α_ radiation (λ = 1.54056 Å). Thermogravimetry curves were obtained using a TG 209 F1 Libra thermo-microbalance (Netzsch, Selb, Germany). The measurement of FTIR spectra was carried out using the IRAffinity-1 spectrometer (Shimadzu, Kyoto, Japan). To carry out photoluminescence studies, the synthesized samples (20 mg) and potassium bromide (300 mg) were pressed into pellets (diameter 13 mm). The photoluminescence data were obtained with a Fluoromax-4 fluorescence spectrometer (Horiba Jobin Yvon, Kyoto, Japan). Lifetime measurements were performed with the same spectrometer using a pulsed Xe lamp (pulse duration: 3 µs). The absolute values of the photoluminescence quantum yields were recorded using a Fluorolog 3 Quanta-phi device (Horiba Jobin Yvon, Kyoto, Japan). All measurements were performed at 25 °C.

## 4. Conclusions

In this study, we explored the structure and the optical properties of brightly luminescent heterometallic terephthalate antenna MOFs, namely, (Eu_x_M_1−x_)_2_(1,4-bdc)_3_·4H_2_O (M = Y, La, Gd) (x = 0.001–1), which were obtained by precipitation from the aqueous solutions. The crystalline phase of all synthesized compounds corresponds to the Ln_2_(1,4-bdc)_3_·4H_2_O (Ln = Ce − Yb) [20]. Unit cell parameters of the obtained compounds were refined by Pawley method. The replacement of Eu^3+^ ions by the larger La^3+^ ions results in an increase in unit cell parameters in (Eu_x_La_1−x_)_2_(1,4-bdc)_3_·4H_2_O compounds, whereas the substitution of Eu^3+^ by smaller Y^3+^ ion results in a decrease in the unit cell volumes in the (Eu_x_M_1−x_)_2_(1,4-bdc)_3_·4H_2_O series. Unit cell parameters almost do not change in the (Eu_x_Gd_1−x_)_2_(1,4-bdc)_3_·4H_2_O series due to close values of ionic radii of Eu^3+^ and Gd^3+^. The dependence of unit cell volume obeys Vegard’s law [24], which allows one to consider the studied systems as solid solutions. All compounds demonstrate a pronounced antenna effect. Upon 300 nm excitation into S_n_ singlet state of the terephthalate ion, obtained compounds demonstrate emission corresponding to the ^5^D_0_–^7^F_J_ (J = 1, 2, 4) transitions of Eu^3+^: ^5^D_0_–^7^F_1_ (587.9 and 591.6 nm), ^5^D_0_–^7^F_2_ (614 nm, maximal intensity), and ^5^D_0_–^7^F_4_ (696 nm). The shape of the emission spectra for the Y-, Gd-, and La-doped compounds is identical to that of pure europium terephthalate emission spectrum due to same local symmetry of Eu^3+^ in the studied solid solutions as a result of crystalline phase isomorphism. The ^5^D_0_ excited state lifetime does not depend on the sample composition and is equal to 0.45 ± 0.03 ms. Meanwhile, the peak intensity of emission spectra depends on the concentrations of europium ions in the studied solid solutions due to different photoluminescence quantum yields. Thus, the peak emission intensities and photoluminescence quantum yields increase with an increase in the Eu^3+^ concentration from 1 to 10 at. % due to the number of luminescence sites increasing. At larger Eu^3+^ concentrations, for the Eu–Y and Eu–La series, PLQY and the peak emission intensities remain the same (about 9–11%), whereas in the Eu–Gd series, they reach a maximum of 15% at the Eu^3+^ content of 10 at. % and then slightly decrease, reaching 10% in pure europium(III) terephthalate. The quantum efficiency of the ^5^D_0_ state was calculated using the relative intensities of ^5^D_0_–^7^F_J_ (J = 1, 2, 4) transitions and ^5^D_0_ excited state lifetimes. To quantitatively compare the antenna effect among different compounds, we propose to use the new approach. Based on the values of PLQYs and quantum efficiency of the ^5^D_0_ state, we calculated the quantum yields of the ^5^D_0_ formation, which reflect the efficiency of energy transfer from the ligand antenna to the Eu^3+^ emitter. The quantum yields of ^5^D_0_ formation are larger for Gd^3+^-doped terephthalates. In the MOFs possessing the highest reported compound values of the PLQY, namely, (Eu_0.1_Gd_0.9_)_2_(1,4-bdc)_3_∙4H_2_O, the value of quantum yield of ^5^D_0_ formation is close to 100%, which indicates a very high efficiency of the energy transfer from the terephthalate antenna to the light-emitting Eu^3+^ ion. The Gd^3+^ ion has the f^7^ configuration and, therefore, the increase in the probability (rate) of S_1_-T_1_ intersystem crossing in the terephthalate ion is associated with the heavy atom effect of paramagnetic Gd^3+^ ions, resulting in the increase in emission intensity, PLQY, and quantum yields of ^5^D_0_ formation.

## Figures and Tables

**Table 1:** The observed ^5^D_0_ lifetime of (Eu_x_M_1−x_)_2_(1,4-bdc)_3_·4H_2_O (M = Gd, La, Y; x = 0.01, 0.1).

Eu_x_M_1−x_	τ(^5^D_0_), ms
Eu_0.01_Gd_0.99_	0.48 ± 0.01
Eu_0.1_Gd_0.9_	0.46 ± 0.01
Eu_0.01_La_0.99_	0.44 ± 0.01
Eu_0.1_La_0.9_	0.43 ± 0.01
Eu_0.01_Y_0.99_	0.47 ± 0.01
Eu_0.1_Y_0.9_	0.45 ± 0.01
Eu	0.44 ± 0.01

## Data Availability

The original contributions presented in the study are included in the article (and Supplementary Material), further inquiries can be directed to the corresponding authors.

## Supplementary Materials

The following supporting information can be downloaded at: https://www.mdpi.com/article/10.3390/molecules29153558/s1, Figure S1: The powder X-ray diffraction (PXRD) patterns of the synthesized compounds series: (a) (Eu_x_Gd_1−x_)_2_(1,4-bdc)_3_·nH_2_O (x = 0.001–1) including the Gd_2_(1,4-bdc)_3_·nH_2_O, (b) (Eu_x_La_1−x_)_2_(1,4-bdc)_3_·nH_2_O (x = 0.001–1) including the La_2_(1,4-bdc)_3_·nH_2_O, (c) (Eu_x_Y_1−x_)_2_(1,4-bdc)_3_·nH_2_O (x = 0.001–1) including the Y_2_(1,4-bdc)_3_·nH_2_O. The positions and relative intensities of diffraction maxima of Eu_2_(1,4-bdc)_3_·4H_2_O taken from ref. [20] are shown as bars, Figure S2: The normalized emission spectra of (a) (Eu_x_Gd_1−x_)_2_(1,4-bdc)_3_·4H_2_O, (b) (Eu_x_La_1−x_)_2_(1,4-bdc)_3_·4H_2_O, and (c) (Eu_x_Y_1−x_)_2_(1,4-bdc)_3_·4H_2_O at a wide concentration range of Eu^3+^ (x = 0.001–1; given in legend) upon 300 nm excitation, Figure S3: The superimposed emission spectra of (a) (Eu_x_Gd_1−x_)_2_(1.4-bdc)_3_·4H_2_O, (b) (Eu_x_La_1−x_)_2_(1.4-bdc)_3_·4H_2_O, and (c) (Eu_x_Y_1−x_)_2_(1.4-bdc)_3_·4H_2_O at a wide concentration range of Eu^3+^ (x = 0.001–1; given in legend) upon 300 nm excitation, Table S1: Unit cell parameters of the (Eu_x_M_1−x_)_2_(1,4-bdc)_3_·4H_2_O (M = Y, La, Gd; x = 0.04–1). Table S2: Eu^3+^ atomic fractions (at. %) in the synthesized compounds, namely, (Eu_x_M_1−x_)_2_(1,4-bdc)_3_∙4H_2_O. Measured data were obtained from EDX.
