# Supplementary material for: The Structure and Optical Properties of Luminescent Europium Terephthalate Antenna Metal–Organic Frameworks Doped by Yttrium, Gadolinium, and Lanthanum Ions
Source: Molecules. 2024 Jul 28;29(15):3558. doi: 10.3390/molecules29153558 (PMC11314237; doi:10.3390/molecules29153558)
Supplement: Supplementary file 1 [file molecules-29-03558-s001.zip › molecules-3113471-supplementary.pdf]

molecules

MDPI

The Structure and Optical Properties of Luminescent Europium Terephthalate Antenna Metal–Organic Frameworks Doped by Yttrium, Gadolinium, and Lanthanum Ions

Oleg S. Butorlin, Anna S. Petrova, Yulia N. Toikka, Ilya E. Kolesnikov, Sergey N. Orlov, Mikhail N. Ryazantsev, Nikita A. Bogachev, Mikhail Yu. Skripkin and Andrey S. Mereshchenko

Supplementary Materials

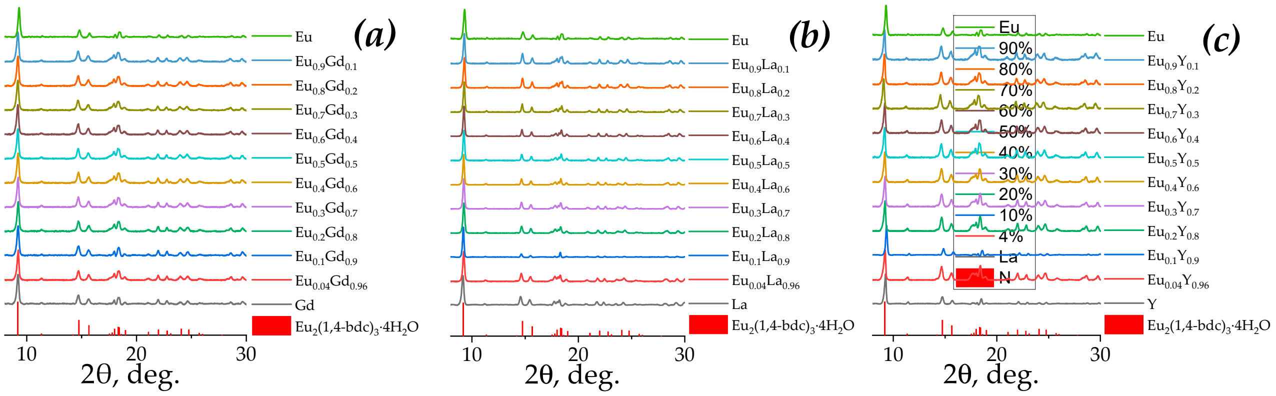

Figure S1. The powder X-ray diffraction (PXRD) patterns of the synthesized compounds series: (***a***) $(Eu_xGd_{1-x})_2(1,4\text{-bdc})_3\cdot nH_2O$ (x = 0.001-1) including the $Gd_2(1,4\text{-bdc})_3\cdot nH_2O$, (***b***) $(Eu_xLa_{1-x})_2(1,4\text{-bdc})_3\cdot nH_2O$ (x = 0.001-1) including the $La_2(1,4\text{-bdc})_3\cdot nH_2O$, (***c***) $(Eu_xY_{1-x})_2(1,4\text{-bdc})_3\cdot nH_2O$ (x = 0.001-1) including the $Y_2(1,4\text{-bdc})_3\cdot nH_2O$. The positions and relative intensities of diffraction maxima of $Eu_2(1,4\text{-bdc})_3\cdot 4H_2O$ taken from ref. [20] are shown as bars.

Table S1. Unit cell parameters of the $(Eu_xM_{1-x})_2(1,4\text{-bdc})_3\cdot 4H_2O$ (M = Y, La, Gd; x = 0.04-1). The parameters obtained within the same series of connections are separated from each other inside the table by a bold line

**$(Eu_xLa_{1-x})_2(1,4\text{-bdc})_3\cdot 4H_2O$**							
**x**	**V, Å³**	**a, Å**	**b, Å**	**c, Å**	**α, °**	**β, °**	**γ, °**
**0.04**	632.6±0.3	6.2732±0.0017	10.251±0.002	10.327±0.003	102.488±0.008	91.988±0.01	101.767±0.009
**0.1**	633.6±0.4	6.272±0.003	10.269±0.003	10.323±0.004	102.433±0.009	92.023±0.012	101.722±0.01
**0.2**	632.0±0.3	6.2653±0.0017	10.261±0.002	10.314±0.003	102.416±0.008	91.988±0.011	101.701±0.009
**0.3**	629.8±0.2	6.2617±0.0014	10.2404±0.0019	10.305±0.002	102.439±0.007	91.829±0.01	101.724±0.008
**0.4**	627.38±0.19	6.2549±0.0012	10.2213±0.0015	10.2910±0.0018	102.410±0.006	91.725±0.009	101.684±0.007
**0.5**	622.74±0.15	6.2375±0.0009	10.1916±0.0013	10.2697±0.0014	102.382±0.006	91.690±0.008	101.637±0.006
**0.6**	620.84±0.15	6.2312±0.0009	10.1781±0.0013	10.2598±0.0014	102.370±0.005	91.617±.008	101.615±0.006
**0.7**	618.49±0.13	6.2230±0.0008	10.1656±0.0011	10.2443±0.0013	102.334±0.005	91.574±0.007	101.598±0.006
**0.8**	615.56±0.13	6.2132±0.0008	10.1468±0.0012	10.2272±0.0013	102.315±0.005	91.518±0.0007	101.546±0.006

**0.9**	613.0±0.2	6.2053±0.0013	10.1384±0.0018	10.2058±0.0019	102.306±0.007	91.476±0.010	101.563±0.008
**1**	610.0±0.2	6.1964±0.0015	10.120±0.002	10.183±0.002	102.233±0.007	91.484±0.011	101.507±0.009
**$(Eu_xGd_{1-x})_2(1,4\text{-bdc})_3 \cdot 4H_2O$**							
**x**	**V, Å^3**	**a, Å**	**b, Å**	**c, Å**	**α, °**	**β, °**	**γ, °**
**0.04**	603.3±0.4	6.173±0.003	10.086±0.004	10.139±0.004	102.197±0.011	91.423±0.019	101.441±0.015
**0.1**	606.2±0.4	6.184±0.003	10.103±0.004	10.152±0.004	102.159±0.010	91.515±0.017	101.419±0.013
**0.2**	604.2±0.4	6.175±0.003	10.094±0.003	10.141±0.004	102.185±0.010	91.465±0.017	101.404±0.013
**0.3**	605.3±0.6	6.180±0.004	10.097±0.005	10.150±0.006	102.195±0.013	91.41±0.02	101.416±0.018
**0.4**	604.2±0.5	6.177±0.003	10.090±0.004	10.142±0.005	102.196±0.011	91.431±0.019	101.438±0.015
**0.5**	605.0±0.6	6.179±0.004	10.098±0.005	10.147±0.005	102.202±0.012	91.45±0.02	101.460±0.016
**0.6**	604.0±0.5	6.178±0.003	10.090±0.004	10.142±0.004	102.205±0.012	91.472±0.019	101.469±0.015
**0.7**	604.7±0.5	6.178±0.003	10.096±0.005	10.147±0.005	102.223±0.012	91.45±0.02	101.471±0.015
**0.8**	604.6±0.5	6.180±0.003	10.091±0.005	10.091±0.005	102.208±0.013	91.50±0.02	101.481±0.017
**0.9**	605.5±0.6	6.183±0.004	10.100±0.005	10.150±0.005	102.215±0.013	91.50±0.02	101.485±0.016
**1**	604.1±0.6	6.184±0.004	10.089±0.005	10.126±0.006	102.114±0.012	91.53±0.02	101.385±0.015
**$(Eu_xY_{1-x})_2(1,4\text{-bdc})_3 \cdot 4H_2O$**							
**x**	**V, Å^3**	**a, Å**	**b, Å**	**c, Å**	**α, °**	**β, °**	**γ, °**
**0.04**	600.1±0.2	6.1767±0.0011	10.060±0.003	10.0942±0.0016	102.016±0.010	91.555±0.014	101.316±0.012
**0.1**	597.2±0.3	6.1659±0.0016	10.043±0.002	10.082±0.003	102.015±0.007	91.555±0.010	101.340±0.009
**0.2**	596.0±0.2	6.1611±0.0015	10.035±0.002	10.078±0.002	102.041±0.007	91.521±0.010	101.349±0.009
**0.3**	598.6±0.4	6.169±0.002	10.054±0.003	10.092±0.004	102.056±0.009	91.520±0.015	101.371±0.013
**0.4**	598.4±0.4	6.166±0.002	10.052±0.003	10.096±0.003	102.066±0.009	91.522±0.014	101.389±0.012
**0.5**	600.0±0.3	6.171±0.002	10.059±0.003	10.110±0.003	102.099±0.010	91.512±0.015	101.405±0.013
**0.6**	601.2±0.4	6.175±0.003	10.067±0.003	10.118±0.004	102.117±0.010	91.479±0.017	101.438±0.014
**0.7**	602.4±0.4	6.177±0.003	10.075±0.003	10.127±0.004	102.125±0.010	91.508±0.017	91.508±0.017
**0.8**	603.2±0.4	6.179±0.002	10.079±0.003	10.133±0.004	102.155±0.010	91.482±0.016	101.444±0.014
**0.9**	604.8±0.4	6.184±0.002	10.091±0.003	10.142±0.004	102.161±0.010	91.492±0.016	91.492±0.016
**1**	604.1±0.6	6.184±0.004	10.089±0.005	10.126±0.006	102.114±0.012	91.53±0.02	101.385±0.015

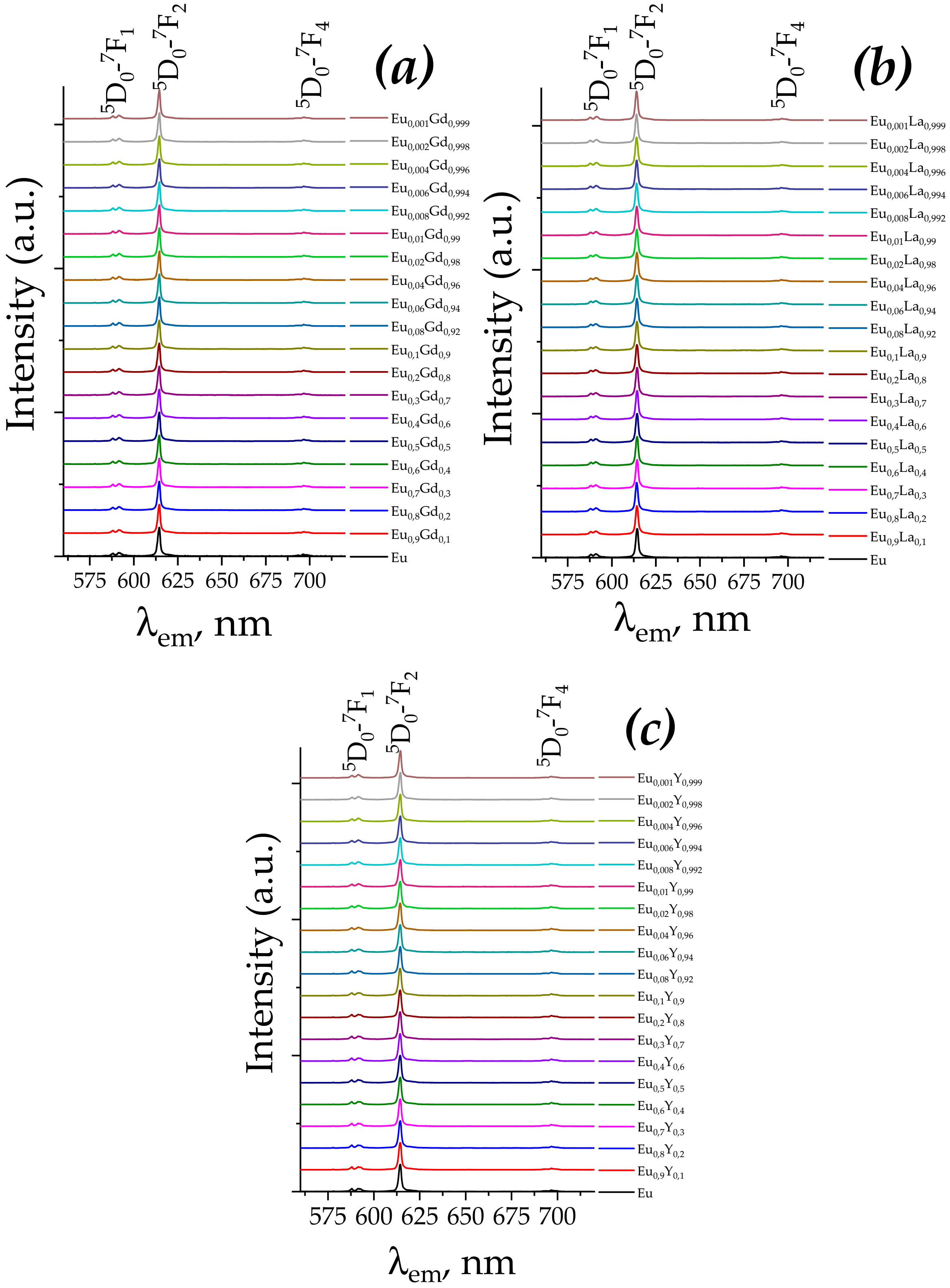

Figure S2. The normalized emission spectra of ***(a)*** $(Eu_xGd_{1-x})_2(1,4\text{-bdc})_3 \cdot 4H_2O$, ***(b)*** $(Eu_xLa_{1-x})_2(1,4\text{-bdc})_3 \cdot 4H_2O$, ***(c)*** $(Eu_xY_{1-x})_2(1,4\text{-bdc})_3 \cdot 4H_2O$ at a wide concentration range of Eu^{3+} (x = 0.001-1; given in legend) upon 300 nm excitation.

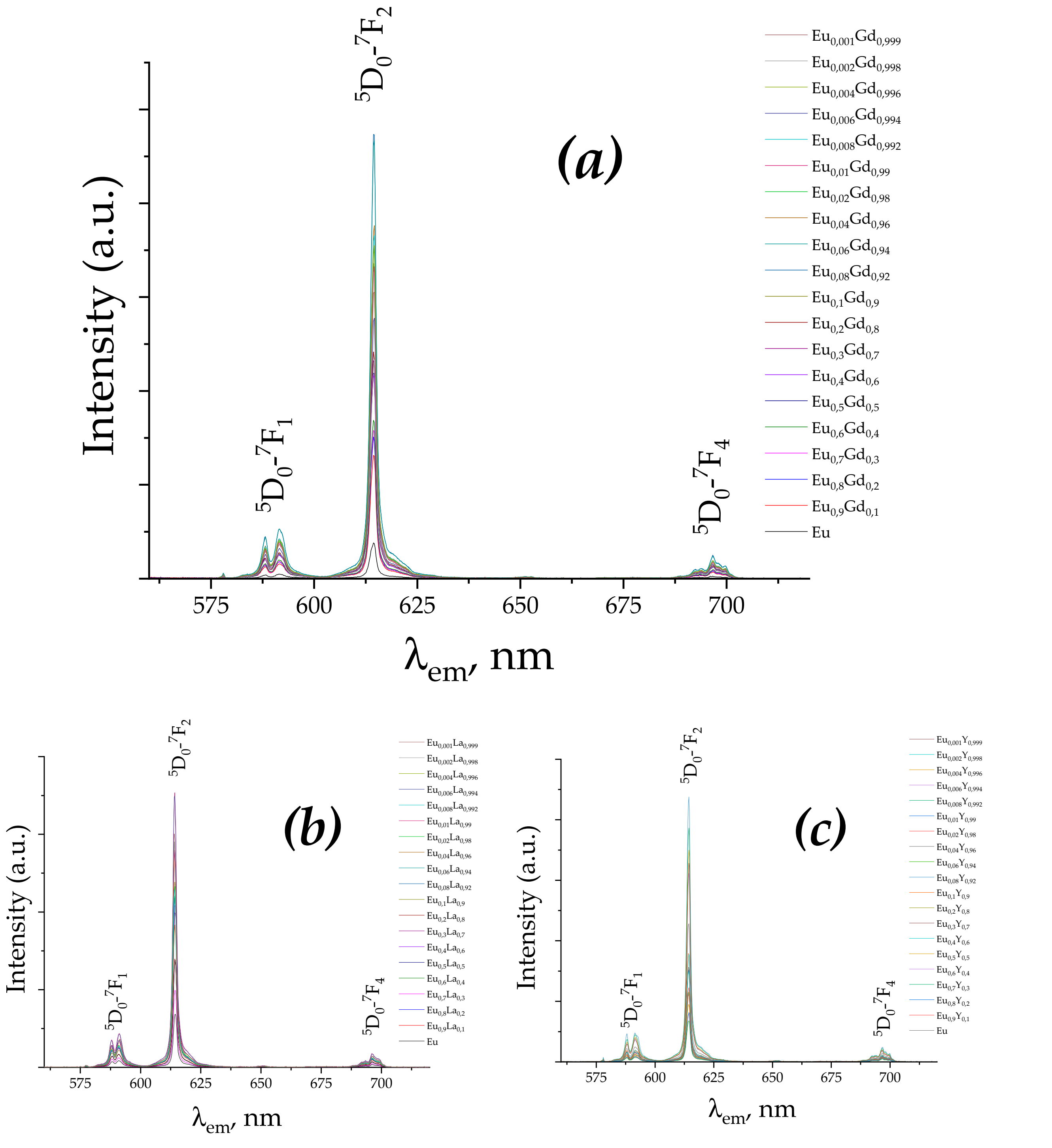

Figure S3. The superimposed emission spectra of ***(a)*** $(Eu_xGd_{1-x})_2(1.4\text{-bdc})_3{\cdot}4H_2O$, ***(b)*** $(Eu_xLa_{1-x})_2(1.4\text{-bdc})_3{\cdot}4H_2O$, ***(c)*** $(Eu_xY_{1-x})_2(1.4\text{-bdc})_3{\cdot}4H_2O$ at a wide concentration range of Eu^{3+} (x = 0.001-1; give in legend) upon 300-nm excitation.

Table S2. Eu^{3+} atomic fractions (at.%) in the synthesized compounds namely $(Eu_xM_{1-x})_2(1,4\text{-bdc})_3{\cdot}4H_2O$. Measured data obtained from EDX.

X_{Eu} (at.%), theoretical	X_{Eu} (at.%) in Eu-Gd series	X_{Eu} (at.%) in Eu-La series	X_{Eu} (at.%) in Eu-Y series
0,1		Low then the LOD*	
0,2		Low then the LOD	
0,4		Low then the LOD	
0,6		Low then the LOD	
0,8		Low then the LOD	
1	2 ± 1	3 ± 1	1 ± 1
2	2 ± 1	3 ± 1	3 ± 1
4	4 ± 1	5 ± 1	5 ± 1
6	7 ± 1	7 ± 1	7 ± 1
8	8 ± 1	9 ±1	8± 1
10	9± 1	11 ± 1	12± 1
20	18 ± 1	24 ± 1	20 ± 1
30	31 ± 2	35 ± 2	31± 2
40	40± 2	44 ± 2	44± 2
50	51 ± 3	55 ± 3	53 ± 3
60	58± 4	63± 4	65 ± 4
70	68± 4	67±4	70± 4
80	85± 5	83 ± 5	83± 5
90	93 ± 5	91± 5	91± 5
100	100	100	100

*LOD – limit of detection.
